# Physicochemical, Structural Structural and Functional Properties of Non-Waxy and Waxy Proso Millet Protein

**DOI:** 10.3390/foods12051116

**Published:** 2023-03-06

**Authors:** Jing Ren, Chao Ma, Mengqing Li, Yueyi Dang, Xiuzhu Yu, Shuangkui Du

**Affiliations:** 1 College of Food Science and Engineering, Northwest A&F University, 22 Xinong Road, Xianyang 712100, China; 2 Shaanxi Union Research Center of University and Enterprise for Grain Processing Technologies, Xianyang 712100, China

**Keywords:** proso millet, protein, functional properties, structure properties

## Abstract

The physicochemical, structural and functional properties of proso millet protein from waxy and non-waxy proso millet were investigated. The secondary structures of proso millet proteins consisted mainly of a β-sheet and ɑ-helix. The two diffraction peaks of proso millet protein appeared at around 9° and 20°. The solubility of non-waxy proso millet protein was higher than that of waxy proso millet protein at different pH values. Non-waxy proso millet protein had a relatively better emulsion stability index (ESI), whereas waxy proso millet protein had a better emulsification activity index (EAI). Non-waxy proso millet protein showed a higher maximum denaturation temperature (T_d_) and enthalpy change (ΔH) than its waxy counterpart, indicating a more ordered conformation. Waxy proso millet exhibited higher surface hydrophobicity and oil absorption capacity (OAC) than non-waxy proso millet, suggesting that the former may have potential applications as a functional ingredient in the food industry. There was no significant difference in the intrinsic fluorescence spectra of different waxy and non-waxy proso millet proteins at pH 7.0.

## 1. Introduction

The emergence of global food security concerns has increased the focus on sustainable food and plant protein sources, which are critical to the overall transformation of food and nutrition security and are currently the focus of discussion in the agro-food arena [1]. As a substitute for animal-derived proteins, plant protein has become the primary source of dietary protein in the majority of developing countries [2,3]. Dietary needs and innovation in food formulation justify the increased consumption of plant proteins to replace animal proteins with a high plant protein content, low cost and sustainability [2,4]. Thus, research on plant protein from proso millet, sorghum and lentils has caused great curiosity among scientists [5].

Proso millet (*Panicum miliaceum* L.) has been a staple food around the world for thousands of years. It is cultivated extensively in Russia, China and India for its short growing season (60–90 days) and its potential for drought and disease resistance, even in harsh and relatively dry climates [6]. In terms of nutrition, the nutritional parameters of proso millet are equivalent to or superior to those of other cereals. The nutrient content of millet is remarkably similar to the recommended ratio of protein, carbohydrate and lipid [7]. The protein content of proso millet is 9.5–17%, which is similar to or higher than that of wheat and other grains. With respect to wheat protein, proso millet protein is enriched in essential amino acids, including isoleucine, methionine and leucine [8]. Additionally, proso millet protein is gluten-free and is thought to protect against liver damage due to its beneficial effects on cholesterol metabolism [9]. Regardless of the many advantages of using proso millet protein, there are still some disadvantages to overcome, including low solubility, poor digestibility and bioavailability [8].

In particular, the application of protein in food depends on its functional properties, which can determine the crucial characteristics of a product [10]. Jarpa-Parra et al. [11] reported that the success of lentil seed protein as a substitute for eggs or milk in angel food cakes is particularly dependent on its ability to serve as a foam stabilizer and emulsifier. According to Mohamed et al. [12], foxtail millet protein has higher trypsin digestibility than soy protein concentrate because it consists primarily of albumin and globulin, which are soluble and easily digested by trypsin. Ravindaran [13] reported an increased digestibility of proso millet protein upon cooking, whereas Kovalev et al. [14] found the opposite. These differences require a further understanding of the quality of proso millet protein.

On the basis of the content of amylose, proso millet can be classified as non-waxy (high amylose content) and waxy (low amylose content) [15]. At present, extensive research work has been conducted on proso millet starch, but there is little research on protein, which affects the further development and utilization of proso millet [16]. Therefore, to facilitate the application of proso millet protein as a functional component in various products, a thorough understanding of how the physicochemical, structural and functional properties of proso millet protein affect its interactions with other biomolecules in the food system is important. Limited research has investigated the differences in the functional and structural properties of different waxy and non-waxy proso millet protein varieties. Therefore, this research aimed to characterize the differences among the physicochemical properties, structure and function of different waxy and non-waxy proso millet proteins cultivars, such as solubility and emulsifying activity properties, via SDS-PAGE, thermal properties and Fourier-transform infrared (FTIR) spectroscopy. This work lays a theoretical foundation for the further in-depth processing and utilization of proso millet protein, with a fundamental reference for its high-quality cultivation.

## 2. Material and Methods

### 2.1. Materials

Four proso millet varieties were obtained from the Agricultural Technology Promotion Center of Shenmu in Yulin, China. Huangmima (N-HMM) and Yumi No.2 (N-YM2) are non-waxy proso millets, and hongruanmi (W-HRM) and Baimi (W-BM) are waxy proso millets. The four proso millet varieties selected for the experiment are typical proso millet varieties cultivated locally in their main production areas. Generally, the two non-waxy proso millet varieties are suitable for processing, such as yellow rice, whereas the two waxy proso millet varieties are suitable for making, such as brewing. All samples were milled uniformly and passed through a 60-mesh sieve. The proso millet powder was defatted with n-hexane by stirring it at room temperature for 2 h. The precipitate was collected and reextracted twice. The defatted proso millet powder was dried overnight at room temperature. The dried samples were stored at −20 °C for protein extraction. All chemicals were of analytical grade.

### 2.2. Preparation of Proso Millet Protein

Proso millet protein was prepared via the isoelectric precipitation method according to Akharume et al. [17], with slight modifications. The defatted proso millet flour was mixed with deionized water at a ratio of 1:8 (*w*/*v*); the pH was adjusted to pH 9.0 with 1 mol/L NaOH and stirred for 2 h at 25 °C. The extract was separated via centrifugation at 7000 r/min for 30 min at 10 °C. The pH of the extract was adjusted to 4.5 with 1 mol/L HCl for protein precipitation and left overnight. Subsequently, the extract was centrifuged at 7000 r/min for 20 min. The proso millet protein (pH 4.5) was washed several times with deionized water in small amounts until the pH was adjusted to 7.0. The precipitate was vacuum freeze-dried (LGJ-18, Beijing Sihuan Scientific Instrument Factory Co., Ltd., Beijing, China) for 24 h and the proso millet protein flour was collected and stored in a refrigerator at −20 °C. Four different varieties of proso millet varieties were divided into four groups, and three replicates were included in each treatment, for a total of 12 groups. Samples were taken from each group for measurements during the experiment.

### 2.3. FTIR Spectroscopy

The secondary structure of protein samples was analyzed via FTIR [18]. A ratio of 1:100 was used to mix the sample and KBr, which was ground into powder and formed into a pellet. The FTIR spectrometer (Vertex 70, BRUKER Inc., Billerica, Germany) was used for full-wavelength scanning (4000–400 cm^−1^), with 32 scans and a spectral resolution of 4 cm^−1^.

### 2.4. Protein Solubility

The solubility in different pH values (3.0, 7.0 and 11.0) was determined according to Akharume et al. [17] with a few corrections. An appropriate amount of protein was dispersed in deionized water to obtain a concentration of 2 mg/mL. The pH of the dispersion was adjusted to 3.0, 7.0 and 11.0 using 0.1 mol/L HCl or 0.1 mol/L NaOH. The suspension was centrifuged at 3000 r/min for 30 min. With proper dilution, the protein content of the supernatant was determined by performing the Bradford method [19] with BSA as the standard. The solubility of the protein was expressed as a percentage of protein present in the supernatant over the total protein content.

### 2.5. Surface Hydrophobicity (S_0_)

The surface hydrophobicity (*S*_0_) of protein samples was determined using the method of Kato & Nakai [20] with slight modifications. The protein samples were obtained through successive dilution in phosphate buffered solution (PBS) (10 mM, pH 7.0) to obtain protein concentrations with a range of 0.36 mg/mL to 0.06 mg/mL. For the concentration of protein samples, 5 mL of each solution was added with 25 μL of 8 mM 1-anilino-8-naphthalene sulfonate solution. After standing in the dark for 15 min, the fluorescence intensity (FI) was determined using a fluorescence spectrometer (F-7000, HITACHI, Hitachi, Japan) at an excitation wavelength of 365 nm and an emission wavelength of 520 nm with a constant excitation and emission slit of 5 nm. The FI was matched linearly with the sample concentration, and the slope of the initial segment was assigned as the *S*_0_ index.

### 2.6. Emulsifying Properties

The emulsifying properties were measured according to the approach of Mohamed et al. [12] with slight modifications. Here, 2 mL of soybean oil was mixed with 6 mL of proso millet protein solution (1%, *w*/*v*) and homogenized (Model T18 Digital, IKA, Billerica, Germany) at 10,000 r/min for 1 min. Then, 50 µL of the homogeneous emulsion was pipetted from the bottom of the container into a test tube at 0 and 10 min. Each sample was mixed with 5 mL of 0.1% SDS solution, and the absorbance of the diluted emulsion was determined at 500 nm. The emulsification activity index (EAI) and emulsion stability index (ESI) were calculated according to the following equations:

(1) $$\mathrm{EAI}\left(m^{2}/g\right)=\frac{2\times 2.303\times A_{0}\times N}{10^{4}\times \varphi \times c}$$

(2) $$\mathrm{ESI}\left(\min\right)=\frac{A_{0}\times 10}{A_{0}-A_{10}}$$

where *N* is the dilution multiple, 100; *ψ* is the oil phase volume ratio, 0.25; *c* is the protein concentration, g/mL; *A*_0_ is the absorbance of the emulsion at 0 min; and *A*_10_ is the absorbance of the emulsion after 10 min of standing.

### 2.7. Water Absorption Capacity (WAC) and Oil Absorption Capacity (OAC)

The WAC and OAC were measured based on the method of Mohamed et al. [12] with slight corrections. For this, 0.5 g of protein was dissolved in a centrifuge tube containing 5 mL of deionized water (or soybean oil) and centrifuged at 3000 r/min for 30 min. The supernatant was removed, and the centrifuge tube with precipitate was inverted on filter paper for 30 min before being weighed. WAC and OAC were expressed as the weight of water and oil adsorbed per gram of proso millet protein, respectively.

WAC and OAC were calculated with the following equations:

$$\mathrm{WAC}\left(g/g\right)=\frac{m_{2}-m_{1}}{m}\;\mathrm{OAC}(g/g)=\frac{m_{2}-m_{1}}{m}$$

where *m*_1_ is the weight of the tube and sample, g; *m*_2_ is the weight of the tube and residue after the adsorption of water (oil), g; and *m* is the weight of protein, g.

### 2.8. Intrinsic Fluorescence

Intrinsic fluorescence was measured as described by Wang et al. [21], with slight modifications. The protein samples were prepared with PBS (10 mM, pH 7.0) at a concentration of 0.2 mg/mL. The intrinsic fluorescence emission spectrum was measured using a fluorescence spectrophotometer (F-7000, HITACHI, Hitachi, Japan) with an excitation wavelength of 295 nm and a scan range of 300–400 nm for the emission spectrum. The slit width was set at 5 nm for excitation and emission spectra.

### 2.9. X-ray Diffraction (XRD)

The crystallographic pattern of the protein was determined via XRD (D8 ADVANCE A25, Bruker, Billerica, Germany) at 40 kV and 100 mA. The samples were first mounted on an aluminum plate and scanner from 5° to 60° (2*θ*) at a rate of 6°/min.

### 2.10. Thermodynamic Properties

The thermal properties of proso millet protein samples were measured using a scanning calorimeter (DSC-Q20, TA Instruments, New Castle, DE, USA), with some modifications [22]. About 3.0 mg of the protein sample was dispersed in 10 μL of deionized water and sealed in an aluminum pan and then allowed to stand at 4 °C for 2 h before scanning. With an empty aluminum pan as a reference, the nitrogen flow rate was 50 mL/min, and the scanning temperature was from 40 °C to 180 °C with a heating rate of 10 °C/min. The DSC thermogram was processed with parameters including the maximum denaturation temperature (T_d_) and enthalpy change of denaturation (∆H). The T_d_ and ∆H were determined, and data were analyzed using TA Universal Analysis 2000 software (TA Instruments Control, New Castle, DE, USA).

### 2.11. Sodium Dodecyl Sulfate-Polyacrylamide Gel Electrophoresis (SDS-PAGE)

The protein profiles were identified through SDS-PAGE following the Laemmli et al. modification [23]. The proteins were analyzed via SDS-PAGE using a 12.5% separating gel and 4% stacking gel. The prepared protein solution (2 mg/mL) was blended with sample buffer containing β-mercapto-ethanol (β-ME) at a ratio of 1:4 (*v*/*v*) under reducing conditions. The mixture was boiled for 5 min and then cooled at room temperature before electrophoresis was performed, with 8 μL of sample loaded into each lane of the gel. The gels were stained using Coomassie blue R-250 and decolored overnight. Electrophoretic profiles were obtained with a Gel Doc XRTM system (Bio-Rad Laboratories, Hercules, CA, USA).

### 2.12. Statistical Analysis

All experiments were measured in three replicates, and the results were expressed as the mean ± standard deviation. ANOVA was performed using SPSS version 26.0 (SPSS, IBM, Chicago, IL, USA). The Duncan’s test at 5% significance level was applied to compare the means.

## 3. Results and Discussion

### 3.1. FTIR Spectrum Analysis

**Figure 1:**
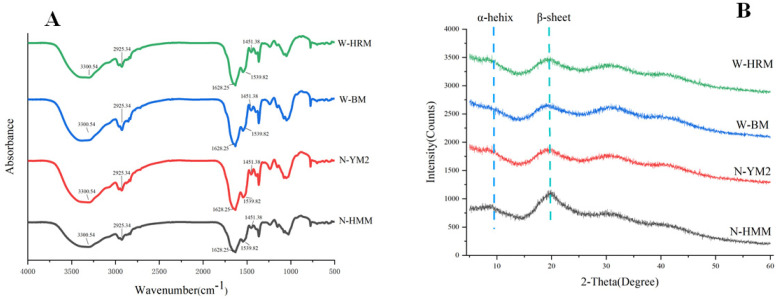
(**A**) Fourier transform infrared (FTIR) spectra and (**B**) X-ray diffraction (XRD) pattern of proso millet proteins. Abbreviations: N-HMM, non-waxy Huangmima; N-YM2, non-waxy Yumi No.2; W-HRM, waxy Hongruanmi; W-BM, waxy Baimi.

FTIR provides vital information about the structure of a protein and its stability. The amide I band (1600–1700 cm^−1^), mainly caused by the C=O stretching vibrations of peptide linkages, was found to be the most sensitive spectral region with respect to the study of the protein secondary structure [24]. Figure 3A clearly shows the characteristic infrared absorption peaks of different waxy and non-waxy proso millet proteins. The protein sample showed a strong and broad absorption peak at 3300 cm^−1^. This peak was due to the O-H bond stretching vibration, which was strong enough to interfere with the N-H stretching vibration absorption peak at a similar peak position. The peak at about 2925 cm^−1^ was classified as the C-H stretching vibration [25]. In general, the basic bonds of the waxy and non-waxy proso millet proteins did not change (Figure 1A).

The absorption peaks of secondary structures (β-sheets, random coils, ɑ-helices and β-turns) where the stretching vibrations were generated were selected for deconvolution. The proportions of β-sheets, random coils, ɑ-helices and β-turns are shown in Table 1. Significant differences were observed in the secondary structure proportions of waxy and non-waxy proso millet proteins (*p* < 0.05). The main secondary structure of waxy and non-waxy proso millet proteins were β-sheets and ɑ-helices, which was consistent with the finding of Wang et al. [26]. More β-sheets were observed than ɑ-helices. By contrast, Ghumman and Kurde observed a higher proportion of ɑ-helices than β-sheets in their research on pulse proteins [26]. The differences in proso millet proteins might be related to the differences in the internal structure. On average, non-waxy proso millet proteins had a higher proportion of β-sheets (54.61 ± 1.13%) compared with waxy proso millet proteins (44.85 ± 2.65%) (*p* < 0.05), suggesting that its structure was relatively highly ordered (Table 1). In addition, the stable thermal properties of the protein could be attributed to the β-sheet conformation, leading to a high T_d_ and ∆H (Table 1) [25]. Conversely, significant differences were observed in the waxy (23.94 ± 1.34%) and non-waxy (14.72 ± 1.48%) proso millet proteins for β-turns (*p* < 0.05). The β-turn ratio is the lowest in the protein secondary structure, and it often improves the conformational stability of the protein by forming a large secondary structure [27].

### 3.2. Protein Solubility

**Figure 2:**
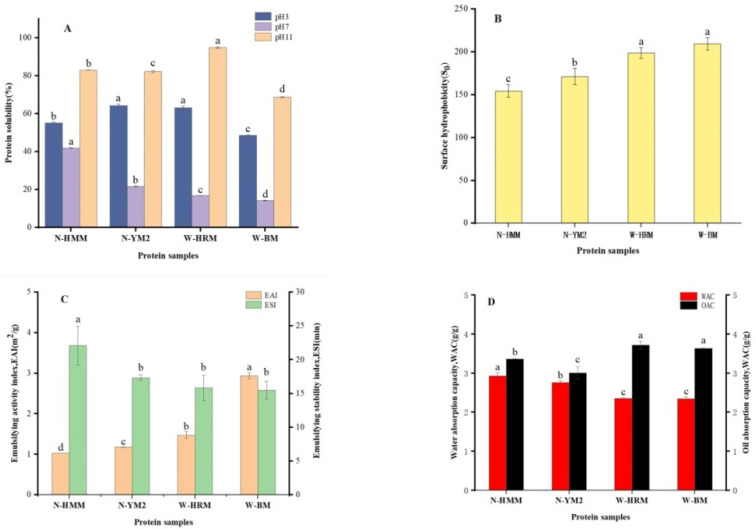
(**A**) Solubility of proso millet protein at various pH values (3.0, 7.0 and 11.0). (**B**) Surface hydrophobicity of proso millet protein at pH 7.0. (**C**) Emulsifying activity properties of proso millet protein at pH 7.0. (**D**) Water absorption capacity and oil absorption capacity of proso millet protein at pH 7.0. Values followed by a different letter in the same column are significantly different (*p* < 0.05). Abbreviations: N-HMM, non-waxy Huangmima; N-YM2, non-waxy Yumi No.2; W-HRM, waxy Hongruanmi; W-BM, waxy Baimi.

The solubility of protein in aqueous solutions influences other functional properties. In general, protein solubility is mainly determined by the balance of hydrophilic and hydrophobic residues in the structure [28]. The solubility of different waxy and non-waxy proso millet proteins at three different pH values (3.0, 7.0 and 11.0) is presented in Figure 2A. On average, the solubility of non-waxy proso millet protein (59.68 ± 4.25%) was significantly higher than that of waxy proso millet protein (55.78 ± 6.76%) at acidic pH (*p* < 0.05) (Figure 2A). A similar increasing trend was shown at neutral and alkaline pH values. The solubility of protein was affected by many factors, such as protein structure and amount of denatured proteins [29]. The relatively low solubility of waxy proso millet protein may be associated with its high surface hydrophobicity (Figure 2B). The low α-helix and β-sheet contents in waxy proso millet protein may counteract the positive contribution of hydrophilic amino acid (AA) residues on the solubility of waxy proso millet protein (Table 1). Protein solubility might be positively correlated with protein structure, such as the α-helix and β-sheet [30], which was in line with the data trend in FTIR (Figure 2A). The solubility peaked under an alkaline pH. The trend in solubility was consistent with the data reported by Mohamed et al. [12]. The proteins are amphiphilic molecules that have both acidic and basic groups. With strong bases or acids, the proteins obtain a net negative and positive charge, which can contribute to molecular repulsion and increase solubility [31].

### 3.3. Surface Hydrophobicity (S_0_)

The *S*_0_ of protein depicts the degree of hydrophobic residue group exposure of the protein molecule and its quantification helps predict protein functions, namely emulsifiability and solubility [17]. The *S*_0_ index of the different non-waxy and waxy proso millet proteins is presented in Figure 2B. The *S*_0_ values of waxy proso millet protein were found to be significantly higher than those of non-waxy proso millet protein (*p* < 0.05). This result was in agreement with the solubility at pH 7.0 (Figure 2A). The low solubility of W-BRM (176.92) and W-BM (209.44) could be attributed to the extensive involvement of hydrophobic residues exposed on the surface of protein molecules at pH 7.0 to participate in intermolecular interactions, resulting in high hydrophobicity and low protein solubility. Similarly, previous research found low α-helix and high β-sheet contents in the protein structure, indicating high surface hydrophobicity [22]. This trend was consistent with the results of FTIR (Figure 1A).

### 3.4. Emulsifying Properties

The ability of proteins to serve as emulsifiers will primarily depend on the physicochemical and conformational properties of the protein [22]. The EAI and ESI of different waxy and non-waxy proso millet proteins at pH 7.0 are shown in Figure 2C. The EAI of waxy proso millet protein (2.21 m^2^/g ± 0.73 m^2^/g) was significantly higher than that of non-waxy proso millet protein (1.10 m^2^/g ± 0.08 m^2^/g) (Figure 2C) (*p* < 0.05). W-HRM and W-BM showed high surface hydrophobicity compared with N-HMM and N-YM (Figure 2B). Higher surface hydrophobicity contributed to a better emulsifying property of proteins [32]. As shown in Figure 2C, the ESI of non-waxy proso millet protein (mean 19.71 min ± 2.37 min) was higher than that of waxy proso millet protein (mean 15.66 min ± 0.17 min). As a result of the high solubility (mean 31.74 ± 9.39%) of non-waxy proso millet protein (Figure 2A), it may provide a high protein distribution at the oil–water interface, thereby forming a thick interfacial layer [33]. Considering the interaction of a large number of hydrophobic amino acids in proso millet protein, oil droplets can be dispersed in the aqueous successive phase of the solution, thereby improving the emulsifying properties of proso millet protein. The above data showed that non-waxy proso millet protein had relatively better ESI, whereas waxy proso millet protein had better EAI. Thus, proso millet with high emulsifying ability can be used as an excellent emulsifier in bakery products.

### 3.5. WAC and OAC

The interactions of water with protein and oil with protein are crucial in the food system because the flavor and texture of the food are influenced by them [17]. The WACs of different waxy and non-waxy proso millet proteins are shown in Figure 2D. The WAC of non-waxy proso millet protein was significantly higher than that of waxy proso millet protein (*p* < 0.05). The WACs of N-HMM and N-YM were 2.93 and 2.76 g/g, respectively, whereas those of W-HRM and W-BM were 2.36 and 2.35 g/g, respectively. Mohamed et al. [12] reported the WAC of yellow millet protein concentrate (6.00 g/g) and white millet protein concentrate (7.00 g/g). This difference in WACs may be due to the grain properties, variations in protein conformations and different separation approaches used [34]. OAC plays a key role in enhancing product taste and flavor retention. The OAC of waxy proso millet protein (mean 3.68 ± 0.04 g/g) was significantly higher than that of non-waxy proso millet protein (mean 3.19 ± 0.18 g/g; *p* < 0.05) (Figure 2D). The high OAC might be partly associated with the higher content of non-polar AA side chains in their protein molecules, suggesting that waxy proso millet protein contains more hydrophobic patches with lipid binding on the protein surface than non-waxy proso millet protein [35]. This observation was in accordance with the results of *S*_0_ (Figure 2B). In summary, proso millet proteins with high WAC and OAC have potential for application in food formulations, such as bakery products.

### 3.6. Intrinsic Fluorescence

**Figure 3:**
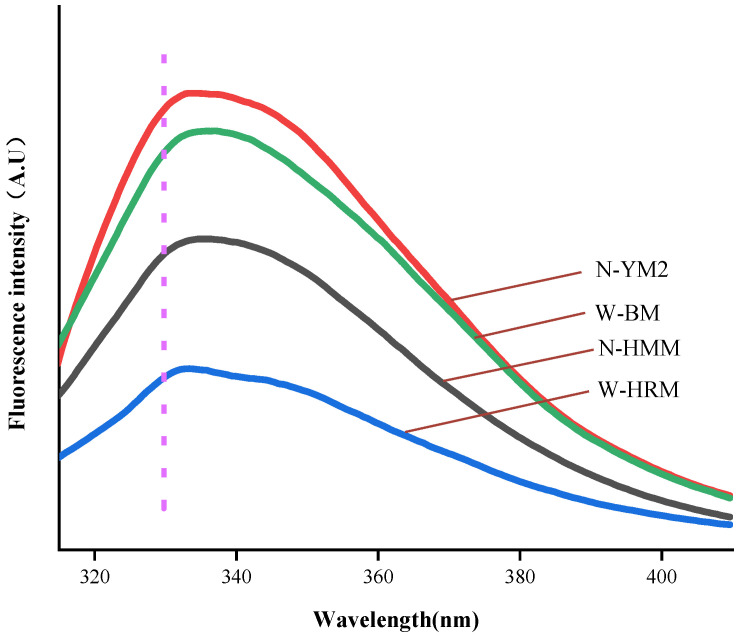
Fluorescence spectra of proso millet protein at pH 7.0. Abbreviations: N-HMM, non-waxy Huangmima; N-YM2, non-waxy Yumi No.2; W-HRM, waxy Hongruanmi; W-BM, waxy Baimi.

The intrinsic fluorescence spectrum of proteins can supply information about changes in protein structure, such as protein folding, unfolding and binding [29]. The fluorescence emission maximum (*λ*_max_) is related to the tryptophan (Trp) microenvironment. Fluorescence spectra of different waxy and non-waxy proso millet proteins at pH 7.0 are illustrated in Figure 3. There was no significant difference in the intrinsic fluorescence spectrum of different waxy and non-waxy proso millet proteins at pH 7.0 (*p* < 0.05). The emission fluorescence spectra of waxy and non-waxy proso millet proteins showed a peak at 334 nm (Figure 3). Typically, Trp is designated as buried and in a “non-polar” environment if Trp fluorescence *λ*_max_ < 330 nm; if *λ*_max_ > 330 nm, the Trp is designated as being in a “polar” environment, which always indicates solvent exposure [36]. The shift in the maximum fluorescence intensity from 330 nm to 334 nm can be clearly observed in Figure 3. The weak red shift of *λ*_max_ suggested an increase in the polarity of the environment surrounding tryptophan that could be attributed to the expansion of the protein structure and the increased contact of the fluorophore with the aqueous medium. Malik et al. [37] also reported that the *λ*_max_ of whey protein isolate had a tiny red shift from 331 nm to 334 nm under treatment at a pH of 12.0. This was also consistent with the data trend reported by Mundi et al. [38] with pH 7.0 treatment.

### 3.7. XRD

The diffraction peaks of different waxy and non-waxy proso millet proteins are shown in Figure 1B, which were similar, but their intensities were different (Figure 1B). Both the non-waxy proso millet protein and waxy proso millet protein showed two diffraction peaks at around 2*θ* = 9° (low intensity) and 2*θ* = 20° (high intensity) (Figure 1B). Malik et al. [37] also found a similar diffraction pattern. According to Jhan et al. [39], two typical diffraction peaks appeared at around 9.0° and 20.0°, which corresponded to the α-helices and β-sheets of the secondary structure conformation of proteins, respectively. Moreover, the intensity of the peak at 2*θ* of 9.0° of non-waxy proso millet protein was higher compared with that of waxy proso millet protein, indicating a higher α-helix content in the former structure, which caused a significant increase in crystallinity (Figure 1A).

### 3.8. Thermal Properties

Through DSC, useful information on the thermal properties of proteins, such as the denaturation temperature (T_d_) and enthalpy change (ΔH) can be acquired. It also can directly reveal the degree of the tertiary conformation of the protein [30]. Table 2 shows the different thermal properties of various waxy and non-waxy proso millet proteins. All protein samples displayed a single enthalpy peak, and the average ranges of T_d_ and ΔH were 87.55 °C ± 2.38 °C and 0.04 ± 0.01 J/g, respectively. Wang et al. (2021) [26] demonstrated that the denaturation temperature and enthalpy changes of rice bran protein isolate were 83.4 °C and 0.96 J/g, respectively. The T_d_ of non-waxy proso millet protein (mean 89.5 °C ± 0.23 °C) was significantly higher than that of waxy proso millet protein (mean 85.61 °C ± 1.65 °C; *p* < 0.05). The T_d_ is related to the AA composition involved, as well as to the protein structure and conformation [26]. N-HMM (89.27 °C ± 0.31 °C) showed higher T_d_ with W-HRM (87.26 °C ± 0.84 °C) and good thermal stability, which might be due to the high ratio of the β-sheet conformation (Table 1). This result was aligned with the FTIR results (Table 1).

The ΔH of non-waxy proso millet protein (mean 0.05 ± 0.01 J/g) was also significantly higher than that of waxy proso millet protein (mean 0.02 ± 0.01 J/g; *p* < 0.05). This finding suggested a more ordered structure in the non-waxy proso millet protein. These values of ΔH were similar to those reported for the globulin (0.50 J/g) and glutelin (0.03 J/g) fractions of proso millet [17]. These values were in agreement with the results of Mohamed et al. [12] reported for yellow millet concentrate (88.98 °C, 0.01 J/g) and white millet concentrate (86.79 °C, 0.10 J/g). Previous research revealed that the ΔH of protein is positively correlated with T_d_; that is, the higher the ordered structure of the protein, the better the thermal stability [30]. In addition, the T_d_ and ΔH in N-YM2 were larger than those of W-HRM. These data suggested that the degree of structural changes of N-YM2 were higher than those of W-HRM, as proven by the intrinsic fluorescence spectra (Figure 3).

### 3.9. Electrophoresis Pattern (SDS-PAGE)

**Figure 4:**
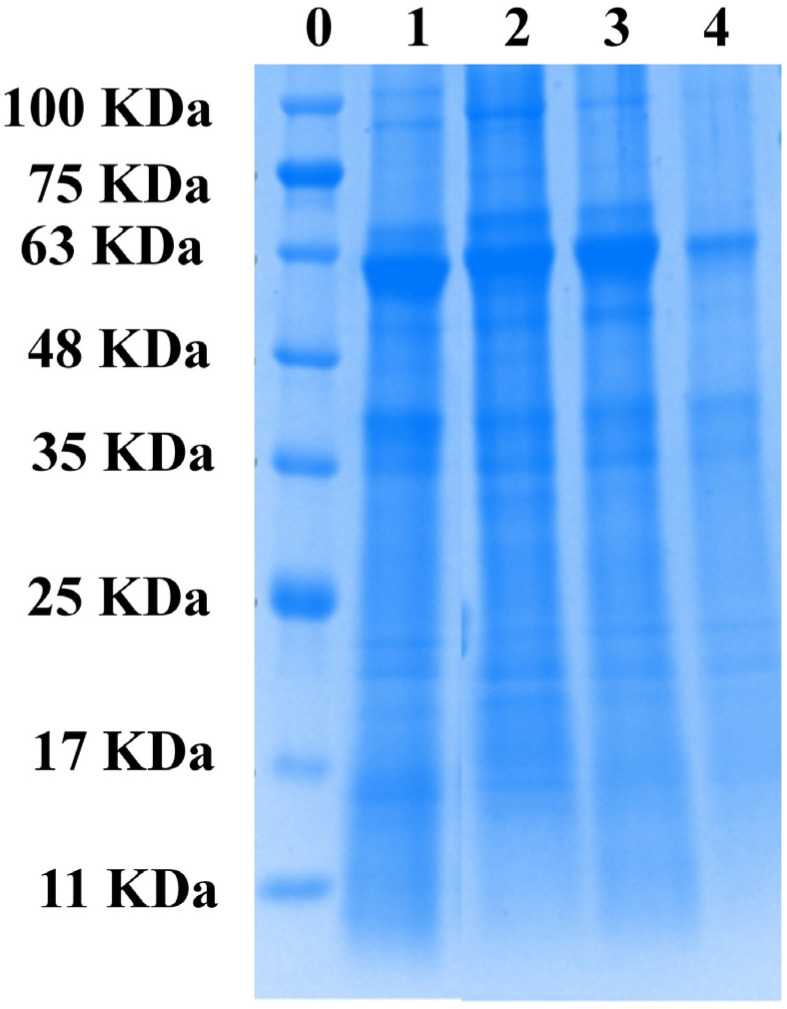
SDS-PAGE patterns of different varieties of proteins. Abbreviations: N-HMM, non-waxy Huangmima; N-YM2, non-waxy Yumi No.2; W-HRM, waxy Hongruanm; W-BM, waxy Baimi. 0-Molecular weight marker, 1-N-HMM; 2-N-YM2; 3-W-BRM; 4-W-BM.

To characterize the composition of proso millet protein, samples were identified via SDS-PAGE. The molecular weights of different waxy and non-waxy proso millet proteins are shown under a reducing condition in Figure 4. A remarkable concordance was observed for the majority of subunits of different waxy and non-waxy millet proteins (Figure 4). The proso millet protein contained about six bands with molecular weights ranging from about 14 kDa to 70 kDa. The molecular weights of the subunits of the major bands were around 69, 60, 54, 40, 33, 30 and 26 kDa. The results were consistent with the research of Wang et al. [26]. However, the amounts of the main subunits of proso millet protein accumulated differently at 60, 54 and 26 kDa, and the accumulation was higher at 60 and 54 kDa. The two distinguishing bands at 60 and 54 kDa clearly observed in proso millet protein were the 7S globulin fraction. According to Zhao et al. [27], peptides with molecular weights of 60 and 54 kDa are the 7S globulin. Proso millet proteins displayed two weak bands at 40 and 26 kDa, which corresponded to the 11S globulin component. These bands were probably ascribed to the combination of these subunits constituting larger protein aggregates. This result agreed with the previous finding that the 11S globulin of SPI consists of two subunits of ~35 and ~25 kDa, respectively [22]. Chihi et al. [40] reported the formation of aggregates in the protein mixture after heat treatment and found that the weakening of the bands may be ascribed to protein aggregation via disulfide bonds.

## 4. Conclusions

Compared with non-waxy proso millet protein, waxy proso millet protein has a higher surface hydrophobicity, oil absorption capacity (OAC), and emulsification activity index (EAI). These results were not unexpected as waxy proso millet protein exhibited relatively low solubility at pH 7.0. The maximum denaturation temperature (T_d_) and emulsion stability index (ESI) of non-waxy proso millet protein were higher than those of waxy proso millet protein. This was attributed to the higher β-sheet ratio of non-waxy proso millet protein, indicating that it had a comparatively highly ordered structure. The secondary structure of proso millet protein is mainly composed of β-sheets and α-helices. Moreover, the two diffraction peaks of proso millet protein appeared at around 9°and 20°, respectively.

In conclusion, these results are essential for enhancing the value of proso millet protein and promoting its utilization in the food industry. As the changes in protein composition and structure during food processing can also affect other functional properties of proteins, such as emulsification and oil absorption capacity (OAC), further research may provide clearer guidance to the food industry and alleviate the problem of bulk food shortages to some extent.

## Figures and Tables

**Table 1:** Secondary structure ratio of different proso millet proteins.

Sample	β-Sheet (%)	Random Coils (%)	ɑ-Helix (%)	β-Turn (%)
N-HMM	55.63 ± 0.22 a	9.91 ± 2.62 b	14.75 ± 2.81 ab	14.16 ± 0.97 b
N-YM2	53.59 ± 0.07 a	13.58 ± 0.65 ab	17.54 ± 3.00 a	15.29 ± 2.40 cb
W-HRM	45.22 ± 2.98 b	11.72 ± 2.77 ab	13.49 ± 0.13 ab	24.94 ± 0.32 a
W-BM	44.47 ± 2.88 b	14.27 ± 1.39 a	12.33 ± 1.34 b	22.94 ± 1.78 a

Data are means ± standard deviations (*n* = 3); values in the same column with different letters are significantly different (*p* < 0.05). Abbreviations: N-HMM, non-waxy Huangmima; N-YM2, non-waxy Yumi No.2; W-HRM, waxy Hongruanmi; W-BM, waxy Baimi.

**Table 2:** Thermal properties of different proso millet proteins.

Samples	T_d_ (°C)	∆H (J/g)
N-HMM	89.27 ± 0.31 a	0.05 ± 0.01 a
N-YM2	89.73 ± 0.25 a	0.05 ± 0.01 a
W-HRM	87.26 ± 0.84 b	0.03 ± 0 b
W-BM	83.96 ± 1.42 c	0.02 ± 0.01 c

Data are means ± standard deviations (*n* = 3); values in the same column with different letters are significantly different (*p* < 0.05). T_d_: maximum denaturation temperature, ∆H: change in enthalpy. Abbreviations: N-HMM, non-waxy Huangmima; N-YM2, non-waxy Yumi No.2; W-HRM, waxy Hongruanmi; W-BM, waxy Baimi.

## Data Availability

No data were used for the research described in the article.
